# Impact of Endocrine Disrupting Pesticide Use on Obesity: A Systematic Review

**DOI:** 10.3390/biomedicines12122677

**Published:** 2024-11-24

**Authors:** Marcelino Pérez-Bermejo, Cristian Barrezueta-Aguilar, Javier Pérez-Murillo, Ignacio Ventura, María Ester Legidos-García, Francisco Tomás-Aguirre, Manuel Tejeda-Adell, Miriam Martínez-Peris, Belén Marí-Beltrán, María Teresa Murillo-Llorente

**Affiliations:** 1 SONEV Research Group, School of Medicine and Health Sciences, Catholic University of Valencia San Vicente Mártir, C/Quevedo nº 2, 46001 Valencia, Spain; javierperezmurillo@mail.ucv.es (J.P.-M.); ester.legidos@ucv.es (M.E.L.-G.); paco.tomas@ucv.es (F.T.-A.); manuel.tejeda@ucv.es (M.T.-A.); miriam.martinez@ucv.es (M.M.-P.); belen.mari@mail.ucv.es (B.M.-B.); mt.murillo@ucv.es (M.T.M.-L.); 2 Department of Nutrition. School of Medicine and Health Sciences, Catholic University of Valencia San Vicente Mártir, C/Quevedo nº 2, 46001 Valencia, Spain; crisbaa@mail.ucv.es; 3 Molecular and Mitochondrial Medicine Research Group, School of Medicine and Health Sciences, Catholic University of Valencia San Vicente Mártir, C/Quevedo nº 2, 46001 Valencia, Spain; ignacio.ventura@ucv.es

**Keywords:** pesticide, endocrine disruptor, overweight, obesity

## Abstract

**Background/Objectives:** Endocrine disruptors are substances capable of altering the functions of the endocrine system. There is evidence that some pesticides can be endocrine disruptors and, among some of their effects, we find alterations in pubertal development and in the function of the thyroid gland, which could be related to a greater tendency of obesity. The aim was to evaluate the evidence from clinical and preclinical studies on the association between pesticides used in agriculture and found in plant-based foods with overweight/obesity. **Methods:** This is a systematic review of articles on the impact of the use of endocrine disrupting pesticides on obesity, conducted according to the PRISMA-2020 guidelines. **Results:** There was evidence that some pesticides, such as chlorpyrifos, pyrethroids, and neonicotinoids, may promote obesity and other anthropometric changes by altering lipid and glucose metabolism, modifying genes, or altering hormone levels such as leptin. Other studies suggest that perinatal exposure to chlorpyrifos or pesticides such as vinclozolin may alter lipid metabolism and promote weight gain in adulthood, whereas other pesticides such as boscalib, captan, thiacloprid, and ziram were not associated with changes in weight. Exposure to pesticides such as vinclozolin may be associated with a higher prevalence of overweight/obesity in later generations. **Conclusions:** The few studies that do not show these associations have methodological limitations in data collection with confounding variables. Further studies are needed to provide more and higher quality evidence to determine the true effect of these substances on obesity.

## 1. Introduction

Obesity is considered to be a worldwide pandemic that leads to an increase in medical costs and thus becomes a public health problem [1]. Initially, obesity was considered as an imbalance between energy intake and energy expenditure [2,3,4], sedentary lifestyle, and genetics [4], although the heritability remains a mystery [5] and the origins are multifactorial [6]. Currently, the obesity epidemic is also associated with the increased production of environmental chemicals [7], also called environmental obesogens [8], used mainly in agriculture, as disease vector control, helping to prevent harmful effects caused by fungi, bacteria, or even pests, using pesticides, insecticides, and herbicides [9], or endocrine disruptors (ED), which interfere in different processes.

An ED is an exogenous substance or mixture of substances that alters the functions of the endocrine system and consequently causes adverse health effects in an intact organism or its progeny [10]. EDs include natural and synthetic substances that are widely present in the environment (water, air, food, pesticides) [11]. These substances increase fat mass and participate in adipogenesis, even leading to metabolic disorders. For this reason, they are also called metabolic disruptors [12].

These chemicals that can act as EDs include polychlorinated biphenyls (PCBs), bisphenol A (BPA), and hexachlorobenzene (HCB) found in some pesticides such as pentatrichloronitrobenzene and pentachlorophenol, triclosan, polybrominated diphenyl ethers (PBDEs), dichlorodiphenyltrichloroethane (DDT), or chlordane. Dichlorodiphenyltrichloroethane, DDT degradation (DDE), and hexachlorobenzene (B-HCH) increase the likelihood of metabolic syndrome [13]. They also increase body mass index [14,15,16], total HDL and LDL cholesterol levels [17], and even abdominal waist index [15,18,19]; the latter adverse effect may also occur with excess malathion [20].

Not only agrochemicals can be obesogenic, but products such as fructose [21,22] and some antidiabetic drugs can behave as such [21]; alcohol, chemotherapeutic drugs, dietary fatty acids, and industrial chemicals are also metabolic chemical disruptors [22].

EDs have been implicated in a variety of adverse effects, including neurotoxicity [23], autism spectrum disorder and developmental delays in children after prenatal exposure [24], impaired behavior, learning, memory, attention, sensation and neurodevelopment [25], depression and anxiety in children [26], reduced fertility in women due to increased polycystic ovarian syndrome or premature ovarian failure, and even breast [27] or vaginal cancer [11,28]. EDs can also affect fertility in men [29], especially in the case of some pesticides [30]. They are also associated with endocrine disruption, causing thyroid dysfunction [31], affecting both iodine uptake and thyroid hormone metabolism [32], and interfering with insulin physiology [31], becoming a risk factor for the development of diabetes [27]. They are also associated with the alteration of corticoid function; EDs can act on glucocorticoid receptors, mineralocorticoids, and steroidogenic pathways. Finally, they are associated with induction of oxidative stress, the alteration of arachidonic acid metabolism, or porphyria [33].

Phytosanitary products are pesticides used to maintain the health of crops. This group includes herbicides, fungicides, insecticides, acaricides, plant growth regulators, and repellents [34]. According to the bibliography of the TEDX list (The Endocrine Disruption Exchange), 512 pesticides may have the capacity to alter the endocrine system [35] and finally, according to the European Commission, it is estimated that there are 162 phytochemicals with endocrine disrupting activity [36]. Thus, the enormous presence of EDs in agriculture and their potential health risk is evident.

Many regions of the planet are currently experiencing water scarcity. This is due to both biophysical factors and factors that increase the demand for water, resulting in an increase in water stress [37]. Wastewater has become an option as a non-conventional water source to alleviate the water demands of the agricultural sector. However, the discharge of untreated or inadequately treated wastewater could have harmful effects on human health, the environment, and economic activity [38]. In rich countries, 70% of wastewater is treated, while in upper-middle-income, lower-middle-income, and poor countries, this figure drops to 38%, 28%, and 8%, respectively [38]. It is estimated that about 10% of the world’s irrigated area uses untreated wastewater [37]. Improper treatment prior to use as crop irrigation can introduce a range of contaminants, including EDs.

Pollution of irrigation water is not only caused by the reuse of wastewater, but also by the agricultural activity itself. Chemigation is a technique for the application of phytosanitary products that consists of incorporating these products dissolved in the irrigation water itself [38]. The problem arises when pesticides persist in crops and food and contaminate the environment (rivers, lakes, groundwater, soil…). Therefore, the presence of these EDs would indicate both the persistence of these substances and their current use despite their prohibition [39].

Although there are countries where these substances have been banned, levels can still be maintained both in the environment and in the body itself due to the excessively long half-life [40], and numerous efforts are being made to reduce the unhealthy effects of the different types of agrochemicals by implementing product designs that do not present high toxicity [9].

There is evidence that some pesticides have endocrine disrupting effects. These effects include alterations in pubertal development and thyroid function [41], which may be associated with increased susceptibility to obesity. If we add that there are reports of food contamination with these substances due to their use in agriculture [42,43,44,45], it is pertinent to know what effects their consumption could have on our health. Therefore, this systematic review aims to identify those pesticides present in foods of plant origin that could have obesogenic effects on the organism.

## 2. Materials and Methods

This systematic review was conducted according to the Preferred Reporting Items for Systematic Reviews and Meta-Analysis (PRISMA) criteria, which was registered in the records of the Open Science Framework (https://osf.io/hcpsg, accessed 26 October 2024). A literature search was conducted in the PubMed and Web of Science databases in April 2024 using the following terms: pesticide, 2,4-dichlorophenoxyacetic acid, cypermethrin, dithiocarbamate, malathion, bifenthrin, dimethoate, fipronil, lambda-cyhalothrin, chlorpyrifos, imidacloprid, pyraclostrobin, DDT, overweight, and obesity. All pesticides included in the search equation are considered endocrine disruptors as set out in the European Commission’s working document defining criteria for the identification of endocrine disruptors in the context of the application of the Plant Protection Products Regulation and the Biocidal Products Regulation [36]. An initial screening was performed by reading titles and abstracts, followed by a review of the full text of eligible studies.

We selected experimental animal studies, in vitro studies, and human observational studies published since 2000 that investigated environmental or prenatal, perinatal, or postnatal dietary exposure to pesticides, in Spanish or English, and that examined the association of such exposure with overweight or obesity and other anthropometric changes, excluding non-original reports, review articles, conference abstracts, editorials, commentaries, and experimental or pilot studies.

The quality of evidence was assessed using the Agency for Healthcare Research and Quality (ARRQ) scale for human studies, the Systematic Review Center for Laboratory animal Experimentation (SYRCLE) scale for animal studies, and the ToxRTool for in vitro studies.

## 3. Results

**Figure 1:**
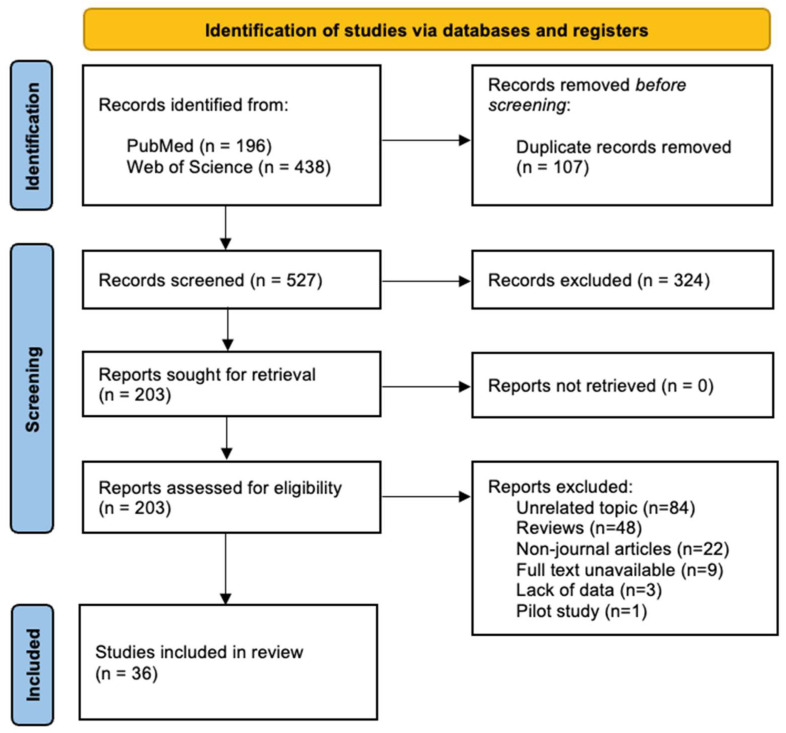
PRISMA flowchart of study selection process.

As shown in the search diagram (Figure 1), 634 articles were retrieved: 196 from PubMed and 438 from Web of Science. After removing duplicates, 527 articles remained. There were 324 articles eliminated after title reading, leaving 203 for full text review. All articles that did not meet the inclusion criteria were excluded. Finally, 36 articles were included in this systematic review: 5 human cross-sectional studies, 24 animal studies, and 7 in vitro studies.

### 3.1. Human Studies

Table 1 shows the five studies selected for this review, all of which were cross-sectional. According to the AHRQ quality rating scale, the quality of evidence of the selected studies was moderate. The studies were conducted in the USA and Thailand. The pesticides evaluated in these studies were 2,4,5-dichlorophenol (2,4-D and 2,5-D), neonicotinoids (imidachlorid, acetamiprid, and clothianidin), glyphosate, diuron, chlorpyrifos, permethrin, mancozeb, maneb, carbendazim, thiophanate, and benomyl.

#### 3.1.1. 2,4-Dichlorophenol and 2,5-Dichlorophenol

The relationship between urinary concentrations of 2,4-D and 2,5-D and weight gain and obesity was evaluated. These studies used data from participants in the National Health and Nutrition Examination Survey (NHANES) [46,47,48]. Two studies evaluated children and adolescents aged 6–19 years [46,48], while the other study evaluated the possible association in adults aged 20–85 years [47]. Participants with obesity were found to have higher urinary concentrations of 2,4-D and 2,5-D. Higher concentrations of these pesticides were associated with increased BMI and waist circumference [46,47,48]. As in children, the adult study showed a higher prevalence of obesity with higher urinary levels of 2,4-D and 2,5-D [47]. However, the study by Noppakun et al. found no significant association between this pesticide and obesity [50].

#### 3.1.2. Neonicotinoids: Imidacloprid, Acetamiprid, and Clothianidin

An association was found between detectable levels of imidacloprid and an 11% higher prevalence of overweight/obesity and a positive association with lean mass index. However, the association was not statistically significant [49]. Similarly, another study did not find a statistically significant association either [50]. However, an inverse association was observed between acetamiprid and fat mass, fat percentage, waist circumference, and BMI. In addition, there was no association between clothianidin and an increase in any measure of adiposity [49].

#### 3.1.3. Other Pesticides

Glyphosate, diuron, permethrin, mancozeb, and maneb were not significantly associated with obesity prevalence. However, carbendazim, thiophanate, benomyl, metalaxyl, propineb, and chlorpyrifos showed a statistically significant association with obesity prevalence [50].

### 3.2. Animal Studies

A total of 24 experimental animal studies were selected for this review (Table 2). All articles were experimental studies evaluating different pesticides and their effects. Of these 24 studies, the following 17 used mice: 13 studies used C57BL/6 mice (7 with the C57BL/6J substrain, 1 with the C5BL/6N substrain and 5 without specifying the substrain), 4 used ICR mice, 1 study used TR apoE3 mice, and 1 used BALB/c mice. The following 7 studies used rats: 3 with Wistar rats, 2 with Long-Evans rats, 1 with Sprague Dawley rats, and 1 did not specify the species.

Of the included studies, 13 used male animals, 4 used female animals, and 7 used mixed populations. The sample sizes ranged from 4 to 133 animals. The studied pesticides were deltamethrin, chlorothalonil, chlorpyrifos, vinclozolin, imidacloprid, bifenthrin, permethrin, lambda-cyhalothrin, malathion, difenoconazole, cyrochlorothalonil, cyrochlorothalonil, vinclozolin, difenoconazole, cyromazine, pirimicarb, chemoclamine, thiram, ziram, glyphosate, metolachlor, thiamethoxam, propamocarb, cypermethrin, boscalid, captan, and thiacloprid. The routes of exposure to these pesticides were gastroesophageal, perinatal, and subcutaneous. The most frequently investigated pesticide was the organophosphate pesticide chlorpyrifos (9 out of 24, 37.5%). For the remaining pesticides, only one study was found, except for imidacloprid, which was found in two studies. In this way, it was possible to observe whether there were changes in appetite, as this could contribute to weight gain and thus to overweight and obesity.

Finally, to evaluate the effect of pesticides on obesity, BMI, waist circumference, weight gain, and fat mass were the main measures used. In addition, several studies assessed biochemical parameters such as insulin levels, blood glucose, serum leptin, thyroid hormones, and lipid profile, as well as biomarkers and indicators of lipid metabolism. All studies were of moderate- to low-quality.

#### 3.2.1. Organophosphates: Chlorpyrifos and Malathion

Chlorpyrifos exposure has been associated with increased body mass, adiposity, and impaired glucose tolerance and insulin sensitivity in male C57BL/6 mice [53,65,67,68,69], CD-1 mice [68], and rats [60] when combined with a high-fat diet. However, this effect was not observed in Wistar rats [64,67]. In addition, a difference was observed between mice with apoE2-TR, apoE3-TR, and apoE4-TR genotypes, with apoE3-TR [65] and apoE4-TR [69] mice showing a greater effect after pesticide exposure. However, this CPF-associated weight gain was not affected by diet type or was not significant [64,68].

Chlorpyrifos has been observed to alter the gut microbiota in C57BL/6 mice, CD-1 mice [68], and Wistar rats [64]. This altered microbiota could have a significant effect on weight gain, serum free lipopolysaccharide concentrations, and fasting glucose. Furthermore, this alteration could promote body weight gain, insulin resistance, and glucose intolerance. In addition, altered intestinal permeability and increased lipopolysaccharide levels were observed, which could induce chronic inflammation and promote the development of insulin resistance and obesity [68]. In addition, chlorpyrifos exposure may alter leptin levels, which could be associated with increased fat deposition, and a strong correlation was found between increased leptin levels and weight gain in CPF-exposed mice [65]. However, another study found that exposure reduced plasma leptin concentrations and had no effect on ghrelin levels [64]. CPF reduced diet-induced thermogenesis and was also observed to alter the synthesis of proteins related to lipid metabolism, with an increase in fat deposition in brown adipose tissue [53].

Similarly, perinatal exposure (during pregnancy) was associated with lower birth weight [66,67] and more rapid weight gain during puberty in males, and males had an altered relationship between body weight and leptin levels in adulthood [58]. Perinatal exposure increased gene expression of lipid metabolism regulatory proteins such as the Srebf1/Srebp1c gene (encoding a protein involved in the regulation of lipogenesis) and the Pparg gene (encoding a protein related to lipid storage) [67]. Malathion exposure has been shown to increase body weight and serum glucose levels in mice [59].

#### 3.2.2. Pyrethroids: Bifenthrin, Permethrin, Lambda-Cyhalothrin, Deltamethrin, and Cypermethrin

The effect of pyrethroids on body weight change has been studied in C57BL/6 mice [51,56,63,72] and in male ICR mice [70]. Deltamethrin was shown to have no significant effect on body mass, adipose tissue (although it was associated with a reduction in fat mass), or insulin tolerance [51]. In contrast, mice exposed to bifenthrin [56] and permethrin [63] had significantly increased body weight, fat mass, and serum cholesterol levels. In addition, permethrin reduced GLUT4 gene expression in muscle [52], whereas the effects of bifenthrin were sex-specific [56].

Exposure to lambda-cyhalothrin [70] and cypermethrin [73] was associated with increased plasma concentrations of free fatty acids, and increased cholesterol was also associated with lambda-cyhalothrin, so it could induce overweight and hyperlipidemia. At the hepatic level, an increase in hepatic lipid accumulation and an increase in PPARγ protein levels (which promote hepatic lipid synthesis) were observed after exposure to lambda-cyhalothrin [70] and a decrease in PPARα (regulators of fatty acid oxidation) after treatment with permethrin [63]. Similarly, cypermethrin treatment was associated with increased hepatic triglyceride levels [73].

#### 3.2.3. Neonicotinoids: Imidacloprid and Thiamethoxam

Exposure to imidacloprid combined with a high-fat diet was observed to cause significant increases in body weight and adipose tissue in male [71] and female [55] mice. Increased epididymal and retroperitoneal adipose tissue in males [71], increased omental adipose tissue in females [55], and increased adipocyte size were observed in both sexes and in males exposed to thiamethoxam. In contrast, thiamethoxam had no significant effect on body weight.

With respect to serum markers, exposure to imidacloprid [71] and thiamethoxam [57] increased levels of TG, free fatty acids, and cholesterol in male mice (in the case of thiamethoxam, there was only a significant change in LDL cholesterol). In females, there were higher TG levels but no significant effects on free fatty acid levels [55]. Imidacloprid exposure was associated with increased serum glucose levels in males and increased insulin and leptin levels in both sexes.

In adipocytes, imidacloprid exposure increased fat accumulation and altered lipid and glucose metabolism, suggesting that it may promote adipogenesis in both sexes. At the hepatic level, thiamethoxam induced tissue damage, fat accumulation, and steatosis. In turn, the effects of imidacloprid and thiamethoxam suggest that it may contribute to a reduction in fatty acid oxidation in the liver [55].

#### 3.2.4. Chlorothalonil

In C57BL/6 and ICR mice treated with low doses of chlorothalonil, there was a significant increase in body weight and in the weight of liver tissue and white adipose tissue. In terms of serum parameters, ICR mice showed an increase in glucose and insulin levels and consequently in the insulin resistance index. In addition, an increase in hepatic lipid accumulation was observed, which was associated with liver damage, and there was an increase in adipocyte size in white adipose tissue [52].

Regarding the effects on the microbiota, an alteration in the intestinal microbiota, both in structure and composition, was observed in mice exposed to the pesticide. This change was associated with an alteration in bile acid metabolism. The study also shows how the change in microbiota may be causally related to obesity in chlorothalonil-treated mice [52].

#### 3.2.5. Vinclozolin

Fetal exposure to vinclozolin in mice was not associated with significant changes. In the F4 litter of exposed mice (transgenerational exposure), a decrease in body weight and a higher prevalence of obesity were observed. Thus, it is suggested that exposure to this pesticide could promote transgenerational epigenetic management of various diseases (including obesity) and sperm epimutations [55].

#### 3.2.6. Difenoconazole

Body weight was significantly decreased in mice exposed to difenoconazole. However, liver weight increased, and increased lipid deposition and hepatic TG levels were observed. At the serum level and glucose and cholesterol levels, both HDL and LDL, were reduced. Thus, it is suggested that it alters glucose and lipid metabolism. However, it is suggested that although it alters metabolism, it does not have an effect that contributes to obesity [61].

#### 3.2.7. Propamocarb

Male mice exposed to propamocarb showed no significant changes in body weight, body weight gain, fat content, or changes in hepatic bile levels. In addition, it was observed that hepatic TGs were reduced, which was associated with an alteration in hepatic lipid metabolism due to propamocarb exposure. Exposure to high doses of propamocarb was associated with metabolic changes related to altered gut microbiota and microbial metabolites [72].

#### 3.2.8. Effect of Pesticide Mixtures

Perinatal exposure to a combination of six pesticides (ziram, chlorpyrifos, thiacloprid, boscalid, thiophanate, and captan) showed no significant differences in body weight and glucose tolerance. An alteration in the activity of the gut microbiota was demonstrated, but this did not show an alteration in body weight gain, subcutaneous adipose tissue, cholesterol levels, or glucose tolerance [74]. The effect of perinatal exposure to a mixture of six other pesticides (cyromazine, MCPB, pirimicarb, quinoclamine, thiram, and ziram) was studied and the offspring showed lower birth weight and reduced insulin and glucagon production. However, in adulthood, these parameters were equal to those of the control group, suggesting a possible capacity for recovery. Finally, an alteration in leptin levels was observed in female rats [62].

### 3.3. In Vitro Studies

We found seven in vitro studies that evaluated the effects of pesticides on the anatomy and physiology of hepatocytes and adipocytes and how such effects may contribute to overweight and obesity (Table 3). The pesticides reviewed in these articles were β-cypermethrin, chlorpyrifos, endosulfan, cis-bifenthrin, fipronil, imidacloprid, quizalofop-p-ethyl, glyphosate, 2,4-D, isoxaflutole, dicamba, quizalofop, and propaquizafop. Only one article was found for each of the pesticides reviewed in the case of endosulfan, fipronil, quizalofop-p-ethyl, dicamba, quizalofop, and propaquizafop. Therefore, the level of evidence in these cases is very low. All articles were rated as reliable without restrictions according to the ToxRTool scale.

#### 3.3.1. Effect on Adipocytes

- β-Cypermethrin

It was observed that exposure of 3T3-L1 adipocytes to β-CYP for 2–4 days had no significant effect on these cells. However, cells exposed to β-CYP exhibited 20% higher levels of reactive oxygen species (ROS) and a reduction in mitochondrial membrane potential (MMP) levels in differentiated adipocytes. These data suggest that the pesticide induces autophagy and adipogenesis by increasing oxidative stress [75].

- Chlorpyrifos

It was observed that exposure of 3T3-L1 preadipocytes to CPF induced a significant increase in the number of differentiated adipocytes and their internal lipid storage capacity. In addition, there was an increase in the expression of PPARγ and C/EBPα transcription factors involved in the function of adipogenesis [81].

- Fipronil

Treatment with the pesticide fipronil significantly increased triglyceride content in adipocytes. It also affected differentiation and lipid metabolism in adipocytes. A significant increase in proteins involved in lipid metabolism and storage was observed in the cytoplasm of adipocytes, resulting in increased lipogenesis and lipid accumulation [76].

- Imidacloprid

Adipocytes treated with imidacloprid showed an increased number of fat droplets compared to the control group. Triglyceride accumulation was increased by 91% and 116% at imidacloprid concentrations of 10 and 20 µM, respectively. Increases in molecular markers related to adipocyte differentiation (CCAAT) and enzymes responsible for lipogenesis (acetyl-CoA carboxylase and fatty acid synthase) were also observed. However, imidacloprid exposure did not affect lipid mobilization in 3T3-L1 adipocytes [77].

- Quizalofop-p-ethyl, glyphosate, 2,4-D, isoxaflutole, dicamba, quizalofop, and propaquizafop

Exposure to QpE for eight days promoted lipid accumulation in adipocytes. However, exposure to QpE metabolites was not able to induce lipid accumulation. Considering that QpE is rapidly metabolized after ingestion, this could indicate that they would not be able to induce lipid accumulation after ingestion. However, in the same study, the commercial formulation of QpE was found to be more potent than the active ingredient. Other pesticides were evaluated in the same study. Treatment with glyphosate, propaquizafop, and 2,4-D showed no effect on adipocytes, whereas dicamba and isoxaflutole increased lipid accumulation [78].

- Fenoxycarb and Pyriproxyfen

It was observed that exposure to fenoxycarb increased lipid accumulation during 3T3-L1 adipocyte differentiation. It also reduced adipocyte viability at high concentrations. An increase in the expression of PPARγ and FATP1, which are involved in fat transport in adipocytes, was induced. In the same study, the effect of pyriproxyfen was evaluated and it was observed that it also induced an increase in lipid deposition, although to a lesser extent than fenoxycarb [79].

#### 3.3.2. Effect on Hepatocytes

- Cis-Hifenthrin

HepG2 cells were exposed to cis-bifenthrin for 24 h and an increase in intracellular triglyceride levels was observed in these cells. It has also been suggested that there may be an alteration in lipid metabolism by cis-bifenthrin due to the increased activation of PXR and PPARγ [80].

## 4. Discussion

In this systematic review on the obesogenic effects of agricultural pesticides, 36 articles were collected, including observational studies in humans and experimental studies in animals and in vitro. The collected articles evaluated changes and alterations in body weight and other anthropometric parameters, as well as metabolic changes that promote fat accumulation and adipogenesis.

The results obtained show that some pesticides could promote the development of obesity, the most studied being chlorpyrifos. However, contradictory results have been obtained in some cases [50,72,74,75], suggesting the possibility of a potential obesogenic effect of some pesticides, but without obtaining significant results.

In some animal studies, because of the methodology developed, the quality of the evidence on the obesogenic potential of some pesticides is still too low to extrapolate the risk to humans. Data have been obtained on the effect of these pesticides in promoting obesity through exposure during pregnancy or following exposure of parents or other progenitors, in addition to their own consumption [54,62,66,74].

Despite the great diversity of results, it is evident that some pesticides might promote obesity by altering lipid and glucose metabolism, modifying genes, or altering hormone levels such as leptin. Notably, some pesticides showed no effect or even a reduction in weight gain and lipid levels, such as propamocarb [72] and difenoconazole [61].

The endocrine-disrupting effects of many pesticides, such as altering estrogenic, androgenic, and thyroid functions, have been observed [4,12,28]. These alterations could have significant metabolic effects, potentially leading to weight gain and early pubertal development, which is associated with increased obesity risk in adulthood [82].

The review highlights several limitations, including the moderate to low level of evidence, the predominance of animal and in vitro studies, and the reliance on cross-sectional observational studies in humans. There are several important limitations to extrapolating results from animal and laboratory studies to humans. These include biological differences, the discrepancy between the controlled conditions of experiments, and the complexity of human reality, genetic variability, and differences in dose and exposure. In addition, ethical issues play a critical role. Although it is essential to complement animal and laboratory studies with human clinical trials to validate the results and ensure their applicability and safety, this is not feasible in this specific case, so human studies are mainly observational. The diversity of samples studied also limits the ability to draw clear conclusions. Additionally, most studies focus on individual pesticides, whereas the combined effects of multiple pesticides, which are commonly found in food, remain under-researched [43,44].

In this review, only two studies have been found that study the effect on health of a mixture of pesticides [62,74] and, in both, the data do not show an effect on obesity. However, more research is needed in this aspect, since there is the possibility of an additive or synergistic action of pesticides. This is also the point of the PAN report, which mentions that the individual effect of pesticides or mixed with other pesticides is not considered [43].

The need for more research on endocrine disrupting pesticides is well documented. The Endocrine Society and the International Pollutant Elimination Network (IPEN) [83] have highlighted the profound threats to human health posed by endocrine disrupting chemicals (EDCs), including pesticides, and have called for urgent action and further research to understand their full impact. Various agencies such as the Environmental Protection Agency (EPA) [84], which has re-established its Endocrine Disruptor Screening Program, and the National Institutes of Health (NIH) [85] have actively discussed new research focused on interventions to reduce exposure to endocrine disruptors, highlighting the continued need for research and public input.

In addition, there is a growing call to address misleading claims about the safety of endocrine disrupting pesticides, such as the European Food Safety Authority’s (EFSA) guidance on the identification of endocrine disrupting substances in pesticides [86] or Pesticide Facts’ claims that current scientific evidence shows that pesticides are not associated with endocrine disruption at relevant levels of exposure [87]. The aforementioned comprehensive report by The Endocrine Society and IPEN [83] calls for urgent action to address these threats. The World Health Organization (WHO) has also recognized EDCs as a public health priority, emphasizing the need for improved regulatory strategies and public education to protect vulnerable populations. The Endocrine Society’s position statement further emphasizes the importance of public disclosure to ensure that people can make informed decisions and be protected from the adverse effects of EDCs [88].

## 5. Conclusions

This systematic review shows the association between pesticide use and a greater tendency to be overweight and obese. While some studies suggest this association, clear and conclusive evidence is lacking. Pesticides such as chlorpyrifos, pyrethroids, and neonicotinoids were associated with weight gain and other anthropometric changes, while others such as boscalib and captan were not.

The findings underscore the urgent need for policy action, further research, and public awareness. From a policy perspective, the findings underscore the need to review regulations governing pesticide use and health effects. Government agencies and regulators need to consider these findings to develop stronger regulations and promote good agricultural practices that protect both the environment and human health.

From a research perspective, there is an urgent need for more studies to obtain reliable results on the relationship between pesticide use and obesity. Long-term exposure effects and the impact of lifestyle factors, such as high-fat diets, on the obesogenic potential of pesticides need to be investigated.

Public awareness is essential to drive change. Educating the public about the potential health risks associated with pesticide exposure can lead to more informed choices and increased demand for safer agricultural practices. This awareness can also put pressure on policymakers to implement stricter regulations and support further research.

## Figures and Tables

**Table 1:** Characteristics of the human studies included in the systematic review.

Author, Year, Country	Type of Study	Age/Sex/Sample	Agent/Source of Exposure	Assessment Parameters	Main Results	Quality Score
Buser MC et al. [46], 2014, USA	Cross-sectional study	6–19 years/ both/*n* = 1298	2,5-dichlorophenol, 2,4-dichlorophenol/ environmental	zBMI, waist circumference and obesity	Positive association between 2,5-D, 2,4-D with BMI, Hip Circumference, and obesity in children and adolescents.	Moderate (III)
Wei Y et al. [47], 2014, USA	Cross-sectional study	20–85 years/ Both/*n* = 2963	2,5-dichlorophenol, 2,4-dichlorophenol/ environmental	Urinary pesticide concentrations, BMI	Urinary concentrations of 2,5-D were associated with obesity, but there was no significant association with 2,4-D.	Moderate (III)
Twum C et al. [48], 2011, USA	Cross-sectional study	6–19 years/ Both/*n* = 6770	2,5-dichlorophenol, 2,4-dichlorophenol/ environmental	BMI	The prevalence of obesity was significantly associated with 2,5-D, but no such association was demonstrated with 2,4-D.	Moderate (III)
Godbole AM et al. [49], 2022, USA	Cross-sectional study	>19 years/ Both/*n* = 1675	Imidacloprid, acetamiprid, clothianidin/ environmental	BMI, % body fat	Acetamiprid was associated with decreased IMG, % fat, Waist Hip Index, and BMI. Imidacloprid was associated with increased rates of overweight/obesity.	Moderate (III)
Noppakun K et al. [50] 2021 Thailand	Cross-sectional study	≥20 years/ Both/*n* = 20,295	Insecticides, herbicides, fungicides, rodenticides, molluscicides/environmental	BMI, waist circumference	Of the 35 pesticides studied, 22 were associated with increased prevalence of obesity.	Moderate (III)

**Table 2:** Characteristics of the animal studies included in the systematic review.

Author, Year, Country	Species/ Age/Sex/ Sample	Compound/Dose/ Route of Administration/ Duration	Treatment	Control	Parameters of Evaluation	Main Results
Tsakiridis E et al. [51], 2023, Canada	Mice C57BL/6J/ 8 weeks/males/ N = 40	Deltamethrin/ 0; 0.10; 1.0 and 10 mg/kg/ orally/7 days	High- or normal-fat diet with Deltamethrin dose	Same diet without exposure to deltamethrin	Weight, fat mass, lean mass, glucose tolerance, and insulin tolerance	Deltamethrin inhibited UCP1 expression, but did not alter markers of thermogenesis or increase development of obesity and insulin resistance.
Meng Z et al. [52], 2023, China	Mice C57BL/6 and ICR/ 5 weeks/males/ N = 40	Chlorothalonil/ 0.2 mg/L/ oral route/12 weeks.	Ad lib feeding and hydration with chlorothalonil dissolved in water	The water was only deionized	TG, LDL, HDL, AST, ALT, glucose, metagenomic DNA from gut microbiota	Chlorothalonil may alter bile acid metabolism and lead to glycolipid metabolic disorders in the liver.
Wang B et al. [53], 2021, Canada	Mice C57BL/6J/ 7 weeks/males/ N = 10	Chlorpyrifos/ 0.5 mg/kg/ oral route/11 weeks.	High- and normal-fat diet and ad lib hydration with chlorpyrifos	Same diet with no exposure to chlorpyrifos	Weight, body composition, glucose and insulin tolerance, food thermogenesis, mitochondrion	Chlorpyrifos affects mitochondrial function and food thermogenesis promoting increased obesity.
Ben Maamar M et al. [54], 2020, USA	Rats Sprague Dawley/ 70–90 days/both	Vinclozolin/ F0 exposed at 100 mg/kg/day/ intraperitoneal route/6 days	Intraperitoneal injection during pregnancy	DMSO injection during pregnancy	BMI, abdominal adiposity, adipocyte size	There was a higher incidence of pathologies in F4, including obesity.
Sun Q et al. [55], 2017, USA	Mice C57BL/6J/ 5 weeks/females/ N = 4–7	Imidacloprid/ 0, 0.06, 0.6, and 6 mg/kg/day/ oral route/12 weeks.	High- or low-fat diet and hydration ad lib with imidacloprid	Same diet with no exposure to imidacloprid	Weight, insulin, glucose tolerance	Mice treated with imidacloprid significantly increased weight, adiposity, and insulin levels.
Wei C et al. [56], 2019, China	Mice C57BL/6/ 2 months/females/ N = 20	Bifenthrin/ 0.6 mg/kg/ oral route/6 weeks.	Fed corn oil with dissolved bifenthrin and standard ad lib diet	Fed on pure corn oil and standard diet ad libitum	Weight, fat mass, adipocyte size, protein expression	Bifenthrin treatment significantly increased body weight and fat mass.
Yang, D. et al. [57], 2023, China	Mice ICR/ 4–6 weeks/males/ N = 30	Lambda-cyhalothrin/ 0.4 and 2 mg/kg/ oral route/22 weeks.	Diet with low and high doses of lambda-cyhalothrin	Same diet without exposure to lambda-cyhalothrin	Indicators of lipid metabolism, lipid profile, weight	Results suggest that TBI may induce obesity, dyslipidemia, and hepatic steatosis.
Lassiter TL et al. [58], 2008, USA	Rats Long-Evans/ 20–100 days/both/ N = 20	Chlorpyrifos/ 1, 2.5, 4 mL/kg/ perinatal/gestation route	F0 fed with rat feed and water ad lib and chlorpyrifos dissolved in oil	Same diet with pure corn oil	Weight, height, volume, BMI, weight/volume ratio, leptin	Exposure to CPF caused weight gain in males.
Simoni-Berra MA et al. [59], 2023, Mexico	Mice BALB/c/ 4 weeks/males/ N = 20	Malathion/ 10 ppm/ oral route/180 days	Ad lib fed with malathion or malathion + probiotics	Same diet without exposure to malathion	Weight, glucose	Low doses of malathion induced an increase in weight and glucose levels.
Meggs WJ et al. [60], 2007, USA	Rats Long-Evans/ 6 months/females/ N = 20	Chlorpyrifos/ 5 mg/kg/day/ subcutaneous route/ 4 months	Daily injection of chlorpyrifos dissolved in DSMO	Pure DSMO injection	Body and organ weight	CPF-treated mice showed significant weight and fat gain.
Zhang H et al. [61], 2022, China	Mice C57BL/6/ 6 weeks/males/ N = 88	Difenoconazole/ 30 and 100 mg/kg/day/ oral route/28 day	Ad lib feeding with difenoconazole dissolved in corn oil	Same diet with pure corn oil	Lipid profile, intestinal permeability, microbiota, hepatic TG levels	Exposure to difenoconazole was associated with increased lipid accumulation in the liver, affected intestinal permeability, and microbiota.
Svingen T et al. [62], 2018, Denmark	Rats Wistar/ 5–6 months/both/ N= 70	Cyromazine, MCPB, pirimicarb, chemoclamine, thiram, ziram/ doses 5, 16 and 37.5%/ intrauterine route/ Pregnancy	F0 with ad lib feeding and pesticides dissolved in corn oil	Same diet with pure corn oil	Glucose tolerance, insulin, weight	Exposure to high doses was associated with gene alteration in adipose tissue. In males, some degree of weight regain was shown.
Xiao X et al. [63], 2018, USA	Mice C57BL/6J/ 3 weeks/males/ N = 4–8	Permethrin/ 50 μg/kg/d/day/ oral route/12 wk.	High-fat and low-fat diet with dissolved permethrin	Same diet without exposure to permethrin	Glucose, insulin, leptin, TG, cholesterol, weight, fat mass	Permethrin treatment significantly increased body weight and fat mass.
Fang B et al. [64], 2018, China	Rats/ 8 weeks/both/ N = 36	Chlorpyrifos/ 0.3 and 3 mg/kg/day/ oral route/9 weeks	High-fat and low-fat diet with different doses of chlorpyrifos	Same diet with pure DMSO	Weight, glucose, lipids, cytokines, and intestinal microbiome	Chronic exposure to chlorpyrifos was associated with the abundance of opportunistic pathogens and bacteria associated with obese and diabetic phenotypes.
Peris-Sampedro F et al. [65], 2015, Spain	Mice C57BL/6N and TR/ 7 months/males/ N = 40	Chlorpyrifos/ 2 mg/kg/day/ oral route/8 weeks.	Mouse feed ad lib supplemented with chlorpyrifos	Same diet without exposure to chlorpyrifos	Weight, diet, lipids, glucose, total cholesterol	There was a relationship between CPF and obesity in apoE3 mice, although this group is already vulnerable to developing obesity when treated with CPF.
Djekkoun N et al. [66], 2022, France	Rats Wistar/ 2 months/females/ N = 16	Chlorpyrifos/ 10 mg/kg/ oral route/5 days	Standard ad libitum diet in F0 with chlorpyrifos	Same diet without exposure to chlorpyrifos	Glycemia, lipid profile, microbiota	Exposure to chlorpyrifos with a high-fat diet induced dysmetabolism and an imbalance of gut microbiota.
GuibourdenchEM et al. [67], 2021, France	Rats Wistar/ 7 months/both/ N = 67	Chlorpyrifos/ 1 mg/kg/day/ oral route/16 weeks.	Administration of chlorpyrifos dissolved in oil and feeding ad lib	Administration of pure oil and same feeding	Weight, lipid profile, glucose, mRNA of proteins related to lipid and glucose metabolism	Maternal exposure to CPF + high-fat diet was associated with metabolic changes in the offspring and altered lipid and glucose metabolism.
Liang Y et al. [68], 2019, China	Mice C57BL/6 and CD1/ 3 weeks/males/ N = 40	Chlorpyrifos/ 5 mg/kg/day/ oral route/12 weeks.	High-fat and normal-fat diet with chlorpyrifos dissolved in corn oil	Same diet with pure corn oil	Glucose, insulin, lipopolysaccharides, weight, microbiota	Chlorpyrifos caused a breakdown of the intestinal barrier, increased lipid entry, a mild inflammatory state, and increased tendency to gain fat.
Guardia-Escote L et al. [69], 2020, Spain	Mice C57BL/6/ both/ N = 133	Chlorpyrifos/ 1 mg/kg/ oral route/5 days	Oral administration of chlorpyrifos with micropipette + high-fat diet	Same diet with placebo	DNA methylation, leptin, growth factor, weight	Postnatal exposure to CPF caused metabolic alterations in adulthood.
Yang D et al. [70], 2021, USA	Mice ICR/ 6–8 weeks/males/ N = 30	Thiamethoxam/ 4 and 20 mg/kg/ oral route/12 weeks	Ad lib oral feeding with oral administration of TMX	Same diet with phosphate-buffered saline in DSMO 1%	Lipid profile, glucose, tissue index	TMX exposure caused dyslipidemia and fatty liver disease.
Sun Q et al. [71], 2016, USA	Mice C57BL/6J/ 5 semester/males/ N = 30	Imidacloprid/ 0.07, 0.7, and 7 mg/kg/day/ oral route/12 weeks	High-fat and low-fat diet plus imidacloprid administration	Same diet without exposure to imidacloprid	Weight, glucose, insulin, leptin, lipid profile	Imidacloprid was associated with weight gain and adiposity.
Wu S et al. [72], 2018, China	Mice ICR/ 5 weeks/males/ N = 32	Propamocarb/ 0.5, 5, and 50 mg/kg/day/ oral route/4 weeks	Ad lib feeding with propamocarb dissolved in water	Same diet, but with deionized water.	Genes related to lipid metabolism, weight, hepatic TGs	Propamocarb exposure altered transcription of hepatic genes responsible for lipid regulation.
Jin Y et al. [73], 2014 China	Mice C57BL/6J/ 3 weeks/males/ N = 45	Cypermethrin/ 50 μg/kg/ oral route/20 weeks	High-energy diet with cypermethrin administration	Basal diet or high-energy diet without cypermethrin	Weight, lipid profile, hepatic TGs	There were no significant changes in weight. Hepatic lipid accumulation and TG content were significantly increased in the CYP-HFD group.
Smith, L. et al. [74], 2020, France	Mice C57BL/6J 8 weeks/both/ N = 30	Boscalid, captan, chlorpyrifos, thiacloprid, and ziram/ 0.25 mg/kg/day intrauterine/ gestational route	F0 ad lib feeding + administration of pesticide mixture	Same diet without pesticide exposure	Weight, blood glucose, adipose tissue, cholesterol, insulin, urinary, and fecal metabolomes	Perinatal pesticide exposure did not affect body weight or energy homeostasis in 6- and 14-week-old mice.

**Table 3:** Characteristics of the in vitro studies included in the systematic review.

Author, Year, Country	Compound/ Concentration/Time	Type of Tissue	Control	Main Results	Conclusions	Quality Index
He B et al. [75], 2020 China	β-cypermethrin/ 25, 50, and 100 μM/ 2, 4 and 8 days	3T3-L1 adipocytes	Treated with DMEM/10% FBS	Treatment with β-cypermethrin increased ROS levels, autophagy, and adipogenesis.	β-cypermethrin promotes adipogenesis.	16-CSR
Sun Q et al. [76], 2016, USA	Fipronil/ 0.1, 1, and 10 μM/ 8 days	3T3-L1 adipocytes	Treated with DMSO	Fipronil was associated with fat accumulation in 3T3-L1 adipocytes and lipogenesis.	Fipronil alters adipogenesis and increases lipid accumulation.	15-CSR
Park Y et al. [77], 2013, USA	Imidacloprid/ 10 and 20 μM/ 8 days	3T3-L1 adipocytes	Treated with DMSO	Imidacloprid treatment enhanced adipocyte lipid accumulation and lipogenesis regulators.	Imidacloprid could alter adipogenesis and increase fat accumulation.	15-CSR
Biserni M et al. [78], 2019, United Kingdom	Quizalofop-p-ethyl, glyphosate, 2,4-D, isoxaflutole, dicamba, quizalofop, propaquizafop/ 0.1, 1, 10, and 100 μM/ 8 days	3T3-L1 adipocytes	Treated with Dexamethasone	Only QpE showed a statistically significant TG accumulation. In the case of Isoxaflutole and dicamba, the effect was smaller.	The lipid-accumulating capacity of QpE suggests a possible obesogenic capacity.	18-CSR
LIM S et al. [79], 2016, Korea	Fenoxycarb, pyriproxyfen/ 5, 10, 25, 50, and 100 μM/	3T3-L1 adipocytes	Treated with DMEM/10% FBS	Fenoxycarb stimulated PPARγ and FATP1 activity and expression in 3T3-L1 adipocytes. Pyriproxyfen increased lipid deposition to a lesser extent.	Fenoxycarb may promote lipid accumulation in adipocytes.	15-CSR
Xiang D et al. [80], 2018, China	Cis-bifenthrin/ 0.001, 0.01, 0.1, and 1 μM/ 24 h.	Hepatocytes	Treated with DMSO	HepG2 cells incubated with bifenthrin showed a significant accumulation of TG.	Pyrethroid-induced toxicity could alter lipid metabolism.	16-CSR
Blanco J et al. [81], 2020, Spain	Chlorpyrifos/ 25, 50, 100, and 200 μM/ 24 h.	3T3-L1 adipocytes	Treated with DMSO	Chlorpyrifos promotes adipogenesis by increasing the number of 3T3-L1 preadipocytes and improving their lipid storage capacity.	Chlorpyrifos may contribute to increased incidence of obesity.	17-CSR

## Data Availability

No new data were created or analyzed in this study.
